# Correlation Between Aneurysm Size and Hemodynamics in One Individual with Multiple Small Intracranial Aneurysms

**DOI:** 10.7759/cureus.683

**Published:** 2016-07-12

**Authors:** Liangder Jou, Gavin Britz

**Affiliations:** 1 Department of Neurosurgery, Houston Methodist Hospital

**Keywords:** aneurysms, hemodynamics, circle of willis

## Abstract

Objective

A large number of cases are needed in the patient-specific modeling of intracranial aneurysms to establish the statistical significance due to individual variation of risk factors that are difficult to account for. However, these risk factors are critical in hemorrhage risk as demonstrated in large clinical studies. Rupture risks for aneurysms in an individual are easier to compare because these aneurysms are under the same physiological environment, and their only differences are the local hemodynamic factors associated with their anatomic locations.

Methods

Eight small aneurysms (< 7 mm) from one individual were analyzed using patient-specific hemodynamic modeling. Four scenarios with different perfusion assumptions were performed to account for the flow rate at two smaller communicating arteries. Wall shear stresses (WSS) at these aneurysms were compared to determine their relationship with the aneurysm size.

Results

Each of the three largest aneurysms is either the most proximal or distal aneurysm in a given artery so that blood pressure does not have a direct influence on aneurysm size. No wall shear stress-derived hemodynamic variables are found to be related to aneurysm size.

Discussion

A study of multiple aneurysms from one individual offers a unique opportunity to examine various hemodynamic factors without selection biases. Aneurysms greater than 4 mm (Group 1) have a higher product of maximum WSS and area of low WSS; aneurysms smaller than 4 mm (Group 2) have a lower product of maximum WSS and area of low WSS. In addition, aneurysm size is linearly correlated with the flow rate at the parent artery in each group.

## Introduction

Hemodynamics plays an important role in the formation, growth, and rupture of intracranial aneurysms [1-3]. Patient-specific aneurysm modeling allows us to assess the hemodynamic influences on rupture risk of aneurysms that cannot be accomplished in simplified idealized geometry [4], and results of these analyses permit an examination of various hypotheses on the role of wall shear stress (WSS) in the formation, growth, and rupture of aneurysms [5]. A hemodynamic analysis of growing aneurysms can reveal the conditions that predispose aneurysms for growth [6]; a comparison of hemodynamic variables between ruptured and unruptured aneurysms provides an insight on the level and pattern of WSS leading to rupture [7-8]. Castro, et al. showed a greater maximum WSS in ruptured aneurysms [9-10]; however, Boussel, et al. observed a lower WSS at the region where aneurysms grew [11]. Other hemodynamic variables have also been studied [8, 12-13].

There has been no consensus on whether the low or high WSS contributes to aneurysm rupture despite international collaboration and considerable improvement of numerical techniques. To fully examine a hypothesis, a large number of cases is needed to avoid selection biases [10, 12]. In addition, any conclusion from a study is valid only from a statistical viewpoint and does not apply to two aneurysms on different individuals because the hemodynamics could be outweighed by other risk factors [14-16]. For example, smoking and hypertension could raise the rupture risk of an individual considerably and compromise the validity of a hemodynamic study. A comparison of bilateral mirrored aneurysms is less biased [17-18], but these aneurysms are not frequently encountered and are often limited to certain anatomic locations.

Multiple aneurysms exist in approximately 25% of patients with intracranial aneurysms [19-20]. Multiplicity raises the risk of subarachnoid hemorrhage; treatment of one aneurysm may also trigger a de novo aneurysm elsewhere [21] or lead to bleeding of another aneurysm. Therefore, understanding of the relative risk of each aneurysm in an individual with multiple aneurysms is critical for aneurysm management and treatment. Eight small aneurysms of various sizes (< 7 mm) were incidentally discovered in one individual, and these aneurysms were located at almost every anatomical location where aneurysms are often expected [22]. We seek to evaluate hemodynamic variables and relate these variables to their aneurysm sizes because the anatomical location is the only factor that distinguishes these aneurysms from each other.

## Materials and methods

A 72-year-old female was identified incidentally with eight small unruptured intracranial aneurysms during MRI workup for dizziness. She was hypertensive and a lifetime smoker with no previous family history of subarachnoid hemorrhage. Her aneurysms ranged from 1.9 to 6.3 mm. in size. Two were located on the left internal carotid artery (ICA), two on the left middle cerebral artery (MCA), one at the tip of the basilar artery (BA), one at the anterior communicating artery (ACOM), one on the right posterior communicating artery (PCOM), and a multi-lobulated aneurysm at the right MCA bifurcation. The three largest aneurysms were successfully embolized by flow diverters without incidents.

**Figure 1:**
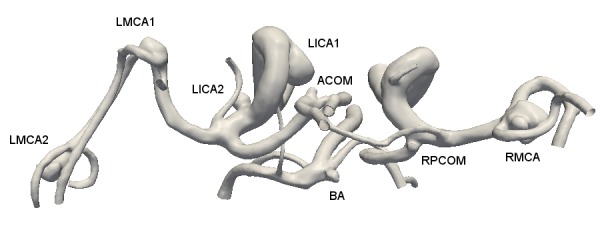
Anatomy of the circle of Willis of the subject and her eight aneurysms.

The 3D rotational angiographic images were obtained by a Siemens AXIOM Artis imaging system (Siemens, Erlangen, Germany), and these images were co-registered together to form the circle of Willis. The subject did not have a complete circle of Willis due to the lack of the right posterior cerebral artery (PCA). The combined images then were processed by Volview (Kitware, Inc., Clifton Park, New York) to create a geometric model beginning from the ICAs and the BA to the distal branches that included all eight aneurysms (Figure 1). The entire model had three flow inlets and 13 outflow branches. The model was further broken down into three smaller parts by removing both the right anterior cerebral artery (ACA) and left PCOM. Each part was analyzed individually and together for the purpose of validation.

### Numerical simulations

The geometric model was meshed by the ANSYS ICEM CFD and flow simulations performed by ANSYS Fluent (ANSYS, Canonsburg, PA). In each of these three smaller models, there was only one major artery for inflow (an ICA or BA). The purpose of smaller models was to evaluate whether the flow was properly resolved in the larger system that demanded a longer computational time and complicated consideration for cerebral blood flow distribution. Each of the three smaller models had 8-11 million cells, and the final system for the entire circle of Willis had 13 million cells after testing for grid independence. 

### Distribution of cerebral blood flow

Because of the numbers of outflow branches and inflow arteries, the following assumptions on the boundary conditions were made. The anterior cerebral circulation was equally distributed between the left and right hemispheres. The ratio of the anterior to posterior cerebral circulation was assumed to be 3:1 [23]; this ratio was based on the physiological flow rates measurements at the ICAs and vertebral arteries (VA) [24]. Thus, the entire model included three territories: one for the posterior circulation, one for the right anterior circulation, and one for the left anterior circulation; blood flows for these territories remained at constant ratios with a total mean cerebral circulation of 12 ml/s [23]. The flow rates at outflow branches were adjusted to achieve a target perfusion rate in each territory.

The flow rate at each branch was adjusted so that flow rates at the outlets met the minimum work principle [25]. Due to hypoplasia of the right PCA, the region that was originally perfused by the right PCA received blood flow from the right PCOM so that the total blood flow to the posterior territory remained the same, but the flow rate at the right ICA was increased accordingly. Flow rates at the two smallest vessels (the left PCOM and right ACA) were adjusted independently.

The flow rates at the ICAs and BA were specified so that each territory maintained a predetermined flow rate described earlier. A waveform measured at the ICA from another female patient using a phase contrast MRA was prescribed at all three inlets, but the waveform at each inlet was adjusted so that the mean flow rate at each region met the perfusion requirement. 

### Data analysis

Aneurysms were organized into two separate groups based on their sizes; three largest aneurysms in Group 1 (> 4 mm and < 6.6 mm) and the other five aneurysms for Group 2 (< 4 mm and > 1.5 mm). In each group, the size range was approximately 2.5 mm. An aneurysm was often referred as tiny when it was less than 4 mm or small when it was between 4 and 10 mm. The time-averaged wall shear stress (TAWSS) and area of low wall shear stress (ALWS) were calculated for each aneurysm. The threshold of the low WSS was set to be 0.4 Pa (pascal) based on an early report [26]. The oscillatory shear index (OSI) was also calculated, along with other variables to distinguish these aneurysms. Each hemodynamic variable (flow rate, TAWSS, ALWS, OSI, etc.) was correlated with aneurysm size by linear regression.

The protocol was approved by a local human subject research committee at our hospital (H-28651) before commencement. Informed patient consent was obtained prior to treatment.

## Results

**Figure 2:**
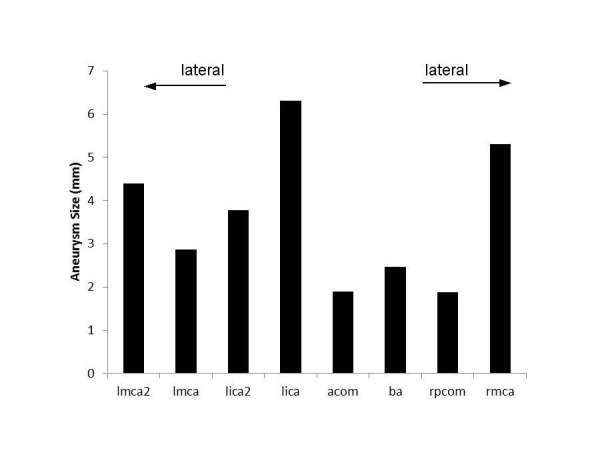
Size distribution of eight aneurysms.

Each of the three largest aneurysms (left ICA1, right MCA, and left MCA2) is either the most proximal or distal aneurysm in a given vessel (Figure 2), and smaller aneurysms are located between them. Since these aneurysms are in the same individual and the distal aneurysms should have a blood pressure approximately 20 mmHg lower than the proximal aneurysms, blood pressure is not found to be related to aneurysm size and higher blood pressure does not lead to a larger aneurysm.

**Figure 3:**
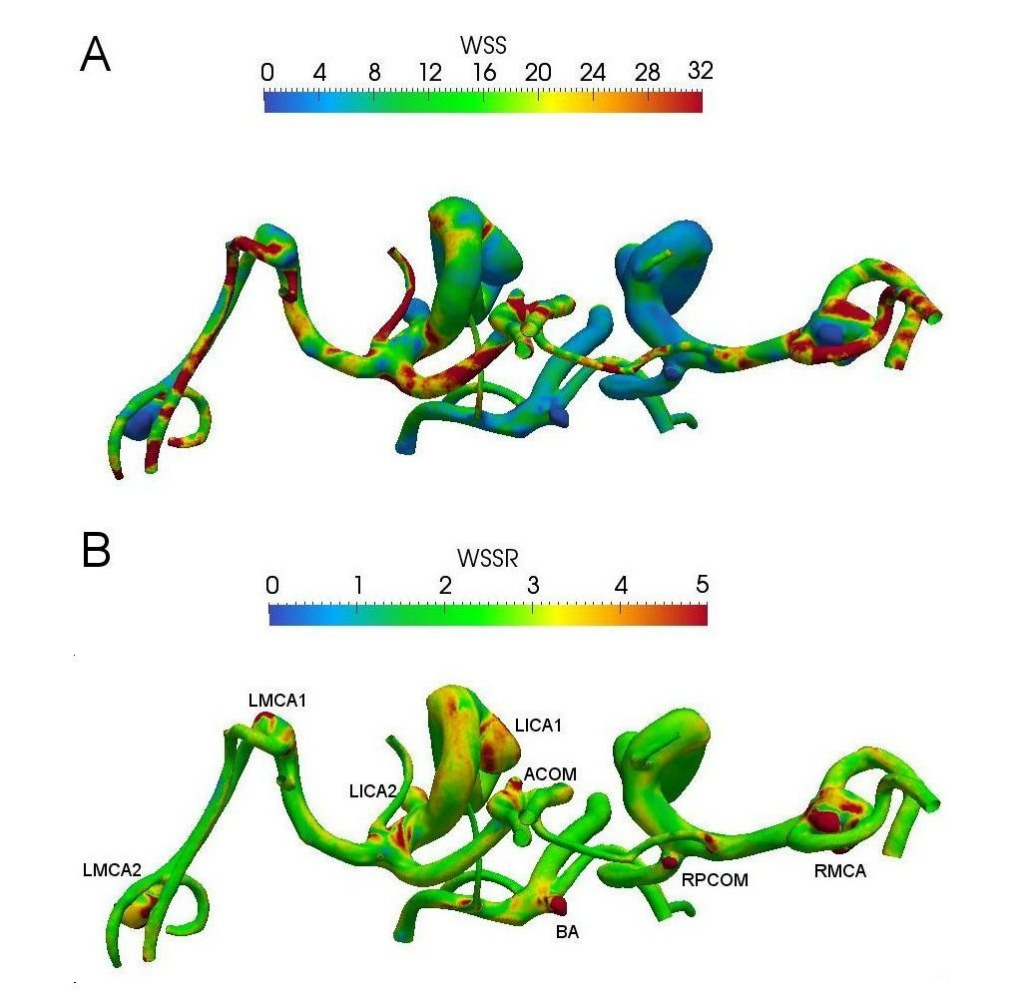
A superoinferior view of WSS distribution for these aneurysms. (A) TAWSS and (B) WSSR (=WSSsystole/WSSdiastole). WSSR represents the level of WSS pulsatility within a cycle.

The TAWSS distribution on the wall is shown in Figure 3A where the maximum TAWSS is found to be at the neck for all aneurysms. The WSS pulsatility (WSSR) is defined as the ratio of the systolic WSS to the diastolic WSS (Figure 3B). The WSSRs at these aneurysms are greater than two, a result of either a greater WSS at the systole or a lower WSS at the diastole. There is no correlation between aneurysm size and the OSI as well.

**Figure 4:**
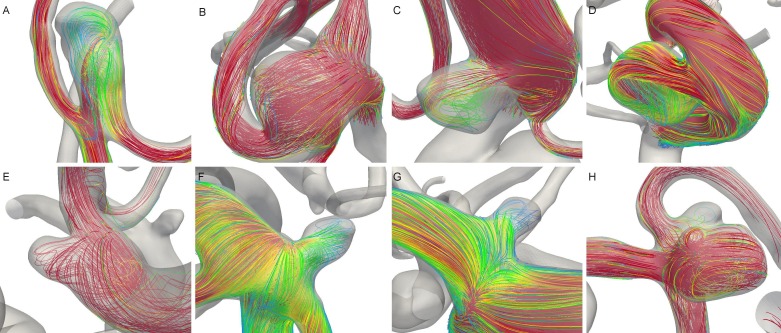
Streamlines at peak systole in these aneurysms. (A) LMCA2, (B) LMCA1, (C) LICA2, (D), LICA1, (E) ACOM, (F) BA, (G) RPCOM, and (H) RMCA.

Intra-aneurysmal flow patterns at the systole for these aneurysms are presented in Figure 4. Two aneurysms at the left ICA exhibit the typical flow pattern in a saccular aneurysm (C and D), while the other aneurysms are terminal aneurysms. A direct flow impingement at the dome is observed only for the LMCA2 (A) and occasionally at the LMCA1 (B). These flow patterns could not explain the difference in aneurysm size as flow impingement occurs only at the distal left MCA aneurysms.

**Figure 5:**
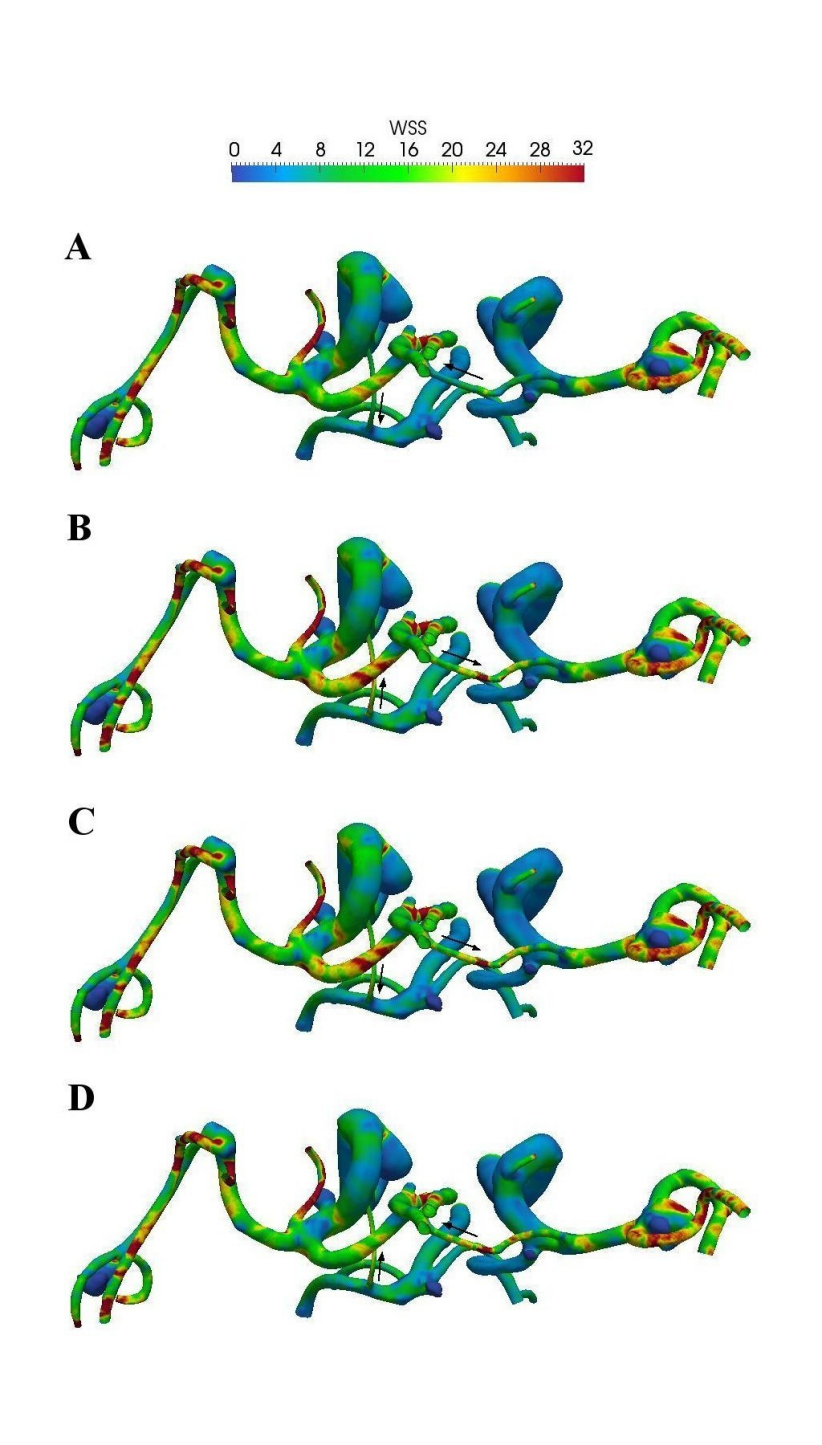
TAWSS distribution based on four different scenarios at two smallest arteries that separate three cerebral territories. Flow directions are indicated by arrows.

Because of the uncertainty of flow direction at the two smallest arteries that divide cerebral territories (the left PCOM and right ACA), four different scenarios are investigated. Figure 5 presents the TAWSS distributions at four different scenarios by altering flow directions at these small arteries. The change of flow direction at these vessels influences the flow rates at the inlets, but the TAWSS at the aneurysms is not significantly different when the flow directions are altered because these two arteries are much smaller than other vessels and carry little blood flow. Changes of the TAWSS are noticeable only for the proximal aneurysm at the left ICA and the aneurysm at the BA; the distal aneurysms are not affected. These changes are likely results of varying flow rates at the inlets and are not associated with aneurysm size.

The size ratio, a ratio of aneurysm size to the parent artery diameter, was found to be related to aneurysm rupture by Meng, et al. [12, 27], and ruptured aneurysms have an average size ratio of 4 versus 2.5 for unruptured aneurysms. In our study, aneurysms in Group 2 have a size ratio between 0.8 and 1.3, and the largest aneurysm in Group 1 has a size ratio of 2.7. However, the size ratio decreases with aneurysm size in Group 1, so the size ratio is not positively correlated with aneurysm size; the narrow size range of our aneurysms is probably responsible for this observation. Nevertheless, Group 1 does have a greater size ratio than Group 2.

**Figure 6:**
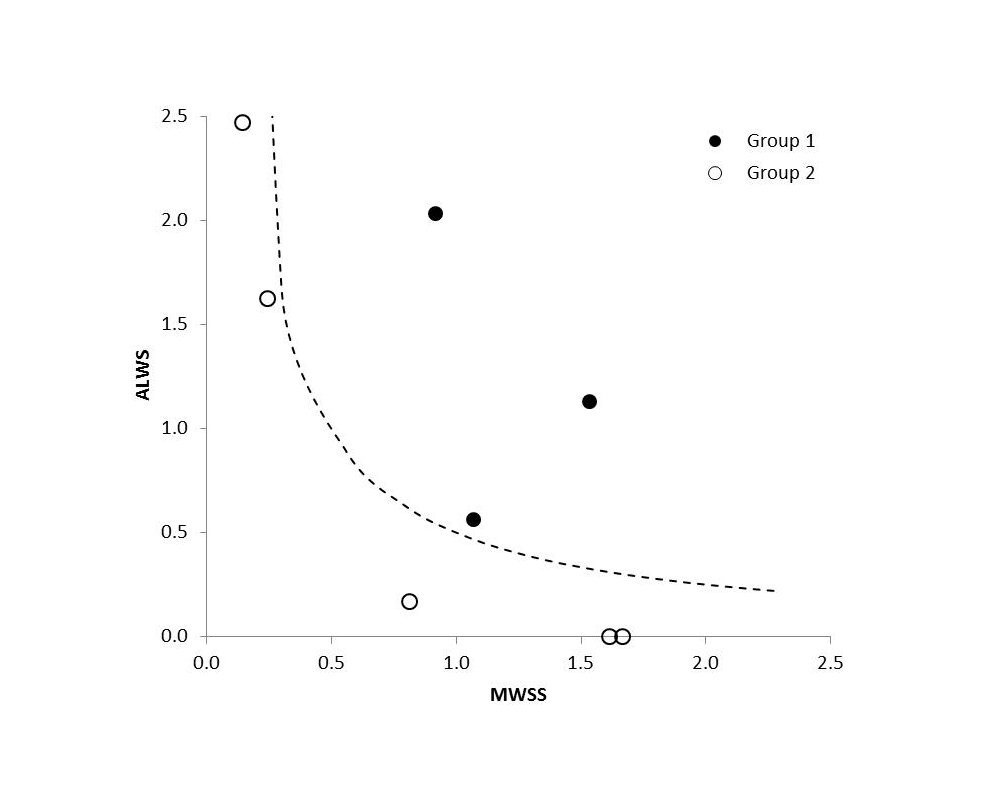
The MWSS versus ALWS. A dashed line (MWSS * ALWS =0.5) separates two groups.

Figure 6 shows a map of the maximum TAWSS and ALWS for these aneurysms. These two variables have been normalized by their average values. The scaling of WSS permits a direct comparison because these two variables have different physical units and physiological meanings. Aneurysms in Group 1 have higher products of MWSS and ALWS (> 0.5), and aneurysms in Group 2 have lower products of these two hemodynamic variables (< 0.25). 

**Figure 7:**
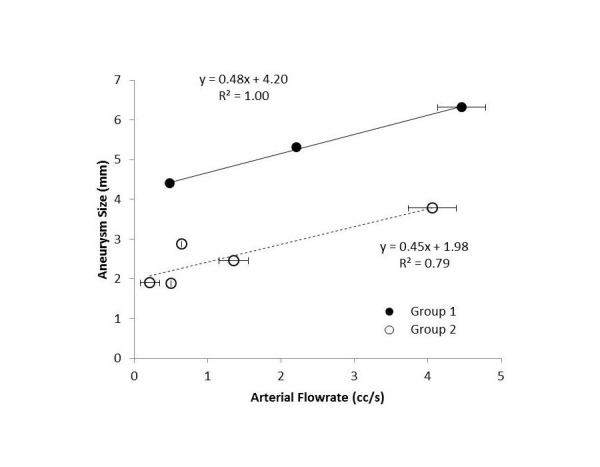
The mean flow rate at the parent artery versus the aneurysm size. The error bars indicate the range of flow rates considered in Figure 5.

A comparison between aneurysm size and flow rate at the parent artery is shown in Figure 7. A higher flow rate at the parent artery does not necessarily lead to a greater MWSS. Data for each group are fitted into a straight line, and these two lines have comparable slopes, indicating a similar influence of flow rate on aneurysm size. In each group, larger aneurysms have higher flow rates at the parent artery, and smaller aneurysms are associated with lower flow rates. This agrees with an observation in an animal study that an aneurysm-like insult was induced by an increase of flow rate [28].

## Discussion

Our study focuses on a unique case where all aneurysms share the same risk factors except for anatomic location. While certain risk factors may have predisposed this patient for aneurysm formation, these risk factors would apply to every aneurysm equally. They differ only in the anatomic location and hemodynamic environment associated with the anatomy, and this is very different from a study for a number of patients in which each aneurysm is subject to a combination of risk factors. Results of our comparison, however, would be difficult to duplicate in studies of aneurysms from a large group of patients. Our approach, nevertheless, is similar to studies of bilateral mirrored aneurysms that compared two aneurysms at a similar anatomic location in the same individual [17-18]. Instead, our study explores the anatomical effect on aneurysm development.

The current study examines the relationship between hemodynamics and aneurysm size so the results should not be confused with the hemodynamic difference between ruptured and unruptured aneurysms. Aneurysms have different levels of TAWSS based on their anatomical locations [29], and two aneurysms with the same level of TAWSS, but at two different locations, are not subject to same rupture risk. Since aneurysms in our study appear at various cerebral arteries, it is not surprising that neither MWSS nor ALWS is correlated with aneurysm size. Chen, et al. found that the TAWSS is the highest for the MCA aneurysms, followed by aneurysms at the ICA, BA, and ACOM [29]; we observed three MCA aneurysms, two ICA aneurysms, a 2.5 mm BA aneurysm, and a 2 mm ACOM aneurysm so the artery with a higher TAWSS does have more aneurysms. The International Study of Unruptured Intracranial Aneurysms (ISUIA) showed that the most prevalent aneurysm locations are the MCA and ICA [22], consistent with our observation in terms of the number of aneurysms at each location.

Our study also reconciles the controversy about the role of WSS on aneurysm rupture [5, 9, 12, 26]. Aneurysms in Group 1 have both a higher MWSS and greater ALWS, but one of these two variables is very low for aneurysms in Group 2. Thus, the concepts of MWSS and ALWS are not mutually exclusive [30]. A higher MWSS does not automatically imply a low ALWS; the theory of high WSS damaging the endothelial cells lining the vessel wall does not contradict with the hypothesis that a vessel wall is weakened by low WSS, and nor does the low WSS hypothesis undermine the impact of high MWSS. It is possible that both factors (MWSS and ALWS) need to be at a certain level to trigger the growth of an aneurysm, and these levels may require special flow patterns previously observed in other studies [9, 30].

There are two possible explanations for the disparity of the group behaviors in Figure 7. First, if all aneurysms form at the same time, then aneurysms in Group 1 grow much faster than those in Group 2. Second, if aneurysms in Group 1 form earlier than those in Group 2 and all aneurysms grow at the same rate, then these two groups differ only in the time of development. Namely, these two groups either have two different growth rates or are at two developmental stages. Aneurysms in Group 1 are either “older” or “growing faster”. In either scenario, aneurysms are influenced by the flow rate at the parent artery, and a higher flow rate in each group creates a larger aneurysm. The slope in Figure 7 then represents the influence of blood flow. Suppose the growth rate is an indication of rupture risk, then aneurysms in Group 1 have comparable risks, even though they are of different sizes. This demonstrates that rupture risk is multifactorial and not a function of one single anatomic or hemodynamic variable, and we cannot rely on aneurysm size or any single variable for assessment of rupture risk.  

Our study is limited by the number of patients presented at our clinic who had a sufficient number of aneurysms. While nearly 30% of aneurysm patients have multiple aneurysms, it is rare for a patient to harbor eight aneurysms and for these aneurysms to be located far enough to represent every possible anatomic location. The majority of patients with multiple aneurysms (> 5) at our institution have most aneurysms at a single artery or near each other, which precludes us from examining the range of flow rate that we have observed in this study. A comparison of aneurysms from different individuals without in vivo measurement of blood flow likely will not be fair.

From examining eight aneurysms in one individual, we have found that there is no single hemodynamic variable that is correlated with aneurysm size. Evaluating rupture risk based on the WSS or any single hemodynamic factor alone is likely to lead to a disappointing result. This highlights the complexity of aneurysm development. Our patient-specific modeling requires certain assumptions, and some assumptions, such as Newtonian fluid and rigid wall, are typical for numerical simulations of this kind. The specified flow rates at the inlets and flow rate ratios at outlets may influence our results. The proposed cerebral perfusion rate for each territory is based on the data from normal volunteers and differs from those of aneurysm patients. However, we have investigated four different scenarios so our observation remains valid despite assumptions of various perfusion rates. 

## Conclusions

Multiple aneurysms in one individual behave similarly to those observed in larger studies on the natural history of cerebral aneurysms, and the difference is that these aneurysms from one individual are subject to the same risk factors, rather than a wide array of inherent and acquired risk factors. Not a single hemodynamic variable based on the WSS is found to correlate with aneurysm size. However, the flow rate at the parent artery is linearly correlated with aneurysm size in both groups. Aneurysms in these two groups also differ in their WSS distribution, with a higher MWSS and greater ALWS for larger aneurysms.
